# Effect of vigorous-intensity exercise on the working memory and inhibitory control among children with attention deficit hyperactivity disorder: a systematic review and meta-analysis

**DOI:** 10.1186/s13052-025-01924-w

**Published:** 2025-03-28

**Authors:** Ruiyun Zhang, Haixia Li

**Affiliations:** 1 School of Sport Art, Shandong Sport University, Lichen District, Jinan, 250102 Shandong China; 2 School of Sport Management, Shandong Sport University, Lichen District, Jinan, 250102 Shandong China

**Keywords:** Vigorous-Intensity, Exercise, Children, Attention deficit hyperactivity disorder

## Abstract

This study primarily evaluated the effects of vigorous-intensity exercises on working memory and inhibitory control in children with attention deficit hyperactivity disorder (ADHD). Search for eligible studies through four databases, and then proceed with screening. The inclusion criteria are as follows: (1) Children with ADHD; (2) Randomised controlled trial; (3) The intervention group received exercise, while the control group did not perform any exercises as the treatment; (4) Conducted pre- and post-exercise assessments, which include working memory and inhibitory control parameters. Use the Cochrane bias risk assessment tool to evaluate the quality of the selected study. Select standardized mean difference as the appropriate effect scale index, and use Revman 5.4 software to analyze the mean difference. This study was registered in the PROSPERO (CRD42024597510). A total of ten studies fulfilled the inclusion criteria and were selected for the meta-analysis. The included studies involved 367 males and 159 females, where 273 belonged to the exercise group and 253 from the control group. Participants in the exercise group enhanced working memory [0.37 (0.12, 0.63) *p* < 0.05, I^2^ = 0%] than the control group. In addition, the results indicated that submaximal intensity exercise improved inhibition regulation levels significantly [− 0.34 (− 0.65, − 0.03), *p* < 0.05, I^2^ = 0%]. Based on the systematic meta-analysis results, vigorous-intensity exercises have effective working memory, cognitive function, and motor ability-increasing effects on children with ADHD. Furthermore, Submaximal intensity exercise can effectively improve control inhibition in children with ADHD.

**Supplementary Information:**

The online version contains supplementary material available at 10.1186/s13052-025-01924-w.

## Introduction

Attention deficit hyperactivity disorder (ADHD) is a significantly prevalent neurodevelopmental illness [1]. The mental ailment is characterised by age-inappropriate inattention, hyperactivity, and impulsivity [2]. ADHD patients predominantly exhibit impaired neurocognitive functioning [3, 4], resulting in various neurocognitive function deficiencies, including attention, inhibition, and working memory [5, 6].

Children suffering from ADHD are commonly prescribed stimulant medication as an effective treatment for reducing behavioural symptoms [7] and improve neurocognitive functioning [8]. Nonetheless, the intervention medication poses several risks, such as sleep issues, decreased appetite, and headaches [9]. The side effects of stimulant medication are unacceptable to some families [10], specifically when involving young children [11]. Moreover, the intervention does not completely improve the attentional, behavioural, and social deficits characterising ADHD [12]. Typically, the symptoms return in the months following treatment, even with the effective implementation of the evidence-based intervention [13].

A potential non-pharmacological option for improving working memory and inhibitory control in children ADHD patients is physical activity. Exercise is defined as any skeletal muscles-induced bodily movements that require energy [14], encompassing numerous forms such as deliberate exercise, sport, play, and active transport. Naturally occurring play activities young children frequently engage in, including tag or chasing games, are some forms of vigorous-intensity aerobic exercises, hence potentially beneficial [15]. Consequently, developing structured interventions focusing on innately appealing and enjoyable recreations to diminish dysfunction and enhance well-being is a promising advantage.

A previous meta-analysis [16] investigated the impact of physical exercise on executive function in children with ADHD, but did not differentiate between different exercise intensity, which may lead to different intervention effects. Furthermore, the impact of vigorous intensity exercise on working memory and inhibitory control in children with ADHD is currently unclear. A study suggested that vigorous-intensity exercises can effectively enhance working memory and inhibitory control in children with ADHD [17], some reports have not recorded significant effects [6, 17]. In light of the disparities observed in the outcomes of prior studies [6, 17, 18], this study adopted a systematic review approach to primarily evaluate the effects of vigorous-intensity exercises on working memory and inhibitory control in children with ADHD. The influences of the exercises on secondary indicators, including cognition, inattention, BMI, and exercise ability, were also discussed.

## Materials and methods

### Data sources and study selection

The protocol for the present meta-analysis review was registered in the PROSPERO database (CRD42024597510) on the 15th of October 2024. Two researchers in this study prepared the search strategy and manuscript, adhering to the preferred reporting items for systematic reviews and meta-analysis (PRISMA) guidelines (Appendix A). During the search phase, articles published between the 1st of October 2004, and the 1st of October 2024, were identified from four electronic databases: EBSCO (*n* = 36), PubMed (*n* = 19), Scopus (*n* = 29), and Web of Science (*n* = 53). The keywords utilised during the procedure were “Children”, “Adolescents”, “Exercise”, “Training”, “Vigorous intensity” and “Obesity.”

**Fig. 1:**
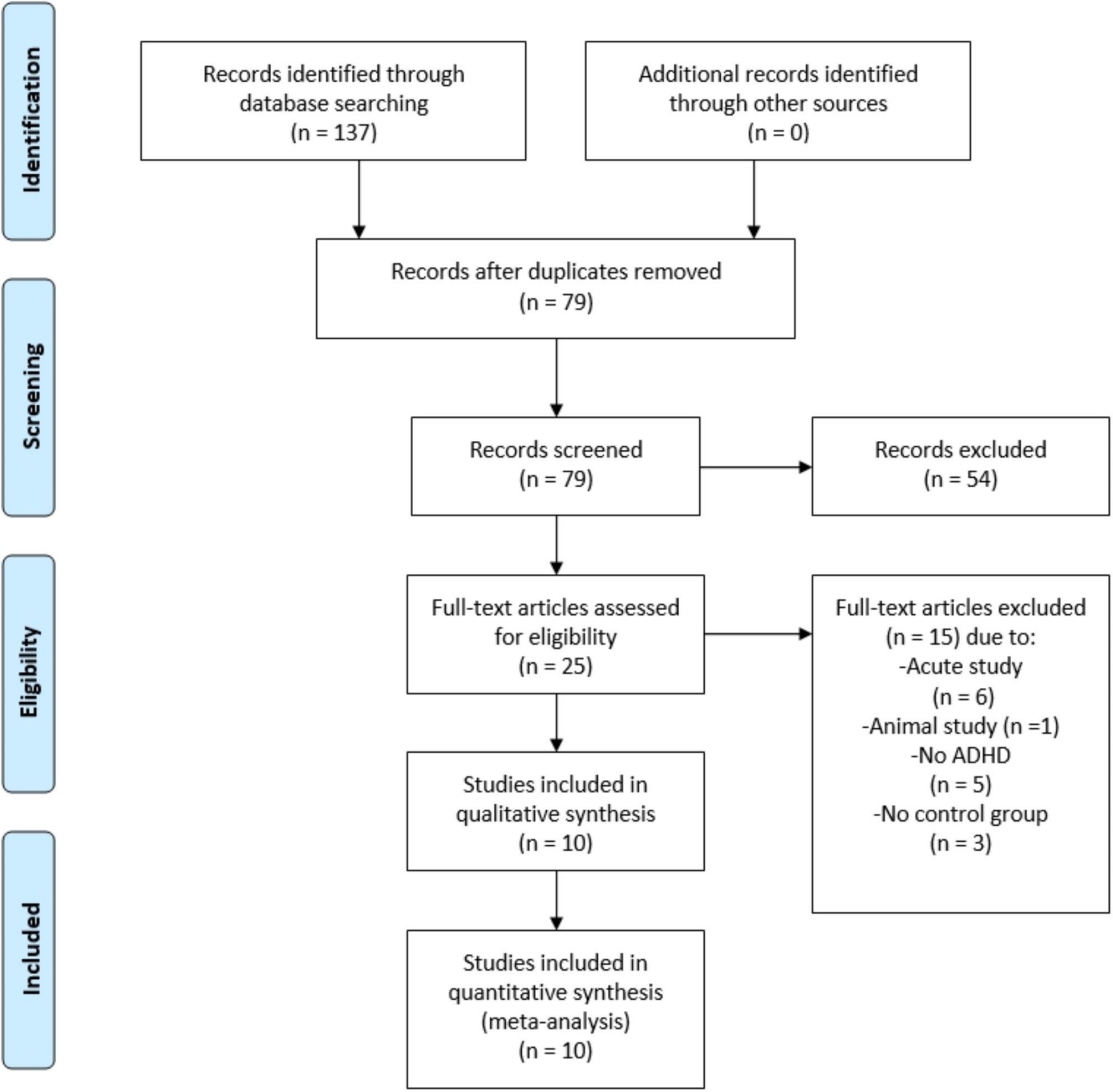
Flow diagram of the search results using the preferred reporting items for systematic reviews and meta-analysis (PRISMA)

Another two independent investigators screened the titles and abstracts of the articles identified in the first phase. Subsequently, the retrieved full publications were re-screened following the inclusion and exclusion criteria. The quality of all articles that fulfilled the inclusion criteria was then assessed before extracting the data. In the event of a dispute concerning the retrieved publications, an independent researcher was asked to consider the matter to attain a consensus. The article selection protocol applied in this review is demonstrated in Fig. 1.

### The inclusion and exclusion criteria

The present study only considered publications meeting six inclusion criteria. Only articles conducting randomised controlled trials, involving participants with ADHD under 14 years old, implemented vigorous intensity exercises as interventions for the experimental group, the participants in the control group did not perform any exercises as the treatment, working memory and inhibitory control were included as evaluation indicators, and utilised English as the language medium were reviewed. Working memory is tested using digital working memory tasks. Inhibition control is achieved by stop-signal task. eligibility criteria This study did not consider abstracts, conference proceedings, and poster presentations.

### Quality assessment

The methodological standard of the articles identified in this review was evaluated based on the Cochrane risk of bias assessment tool [19]. The instrument determined several biases, including random sequence generation, allocation concealment, participants and personnel blinding, outcome assessment blinding, incomplete outcome data, and selective reporting [19]. Each item was assigned a “Yes”, “No”, or “Unclear” score to describe the quality of the reports evaluated.

### Data extraction

Table 1 summarises the details from each selected study, including age, sample size and gender, duration, frequency, exercise program, and index. The data extraction process was performed independently by two co-reviewers. Another researcher was involved during disagreements.

**Table 1:** Characteristics of included studies

Study	Age(y)	Gender	Duration	Frequency	EXE protocol	Index
Benzing 2019	10.6 ± 1.3	43 M/8F	8 weeks	3x/week	Game, vigorous intensity, 30 min	IC, Motor ability
Bustamante 2016	9.1 ± 2.1	23 M/11F	10 weeks	3x/week	Game, 75-103%HR_max_, 90 min	Inattention, IC, WM
Gelade 2016	9.6 ± 1.8	53 M/16F	12 weeks	3x/week	HIIT, 80–100%HR_max_, 2 min×2 min, 5 sets	Inattention, IC, WM
Hoza 2014	6.8 ± 1.0	50 M/44F	12 weeks	5x/week	Game, vigorous intensity, 31 min	Inattention, Motor ability
Huang 2024	9.56 ± 1.05	27 M/12F	8 weeks	2x/week	Rope skipping, 64–95%HRmax, 30 min	Motor ability, WM
Liang 2022	8.5 ± 1.5	61 M/17F	12 weeks	3x/week	Aerobic and neurocognitive exercise program, 60–80%HR_max_, 60 min	Cognition, IC, WM
Memarmoghaddam 2016	8.3 ± 1.3	36 M	8 weeks	3x/week	Game, 65–80%HRR, 90 min	Cognition, IC
Soori 2019	12.5 ± 0.3	20 M/23F	6 weeks	3x/week	HIIT, 85%HR_max_, 20 m running, 30s interval, 6 sets	BMI
Sun 2024	10.1 ± 1.8	24 M/8F	8 weeks	2x/week	HIIT game, 80–100%HR_max_, 5 min×3 min, 4 sets	BMI, Cognition, IC, Motor ability, WM
Torabi 2017	12.7 ± 1.1	30 M/20F	6 weeks	3x/week	HIIT, 85%HR_max_, 20 m running, 30s interval, 6 sets	BMI, Motor ability

M = Male; F = Female; EX = Exercise; CU = Curcumin; HR_max_= Maximum heart rate; HRR = Heart rate reserve; HIIT = High intensity interval training; BMI = Body mass index; WM = Working memory; IC = Inhibitory control

### Data analysis

All relevant outcome variables identified in this review were entered into the Review Manager (Version 5.4.1, Copenhagen: The Nordic Cochrane Center, The Cochrane Collaboration, 2020) for meta-analysis. Although the selected articles applied continuous variables, the methods and test units employed were different. Accordingly, this study utilised standardised mean difference (SMD) as the index of effect scale.

The current study employed the I [2] statistics to evaluate the heterogeneity between the identified publications. Articles scoring I [2] values under 50% indicated no heterogeneity, hence were analysed with a fixed-effect model. Meanwhile, heterogeneity between the articles was indicated by I [2] figures equal to or over 50%, requiring a random effect model during assessments [20]. This study also performed sub-group analysis to further determine the heterogeneity between the selected articles. Moreover, publication bias was assessed with a funnel plot, while a Forest plot was employed to establish SMD with 95% confidence intervals (CI).

## Results

### Eligibility of studies

This study systematically reviewed 10 randomised controlled trial (RCT) articles [6, 14, 17, 18, 21–26] meeting all inclusion criteria set. The method in each selected article has obtained ethical approval from the respective institutions. The two independent reviewers tasked with screening also reported substantial consistency levels (Kappa coefficient = 0.92). Of 526 participants in the identified publications, 367 were males and 159 were females. A total of 273 and 253 patients were divided into exercise (EXE) and control (CON) groups, respectively. The highest exercise intensity range values in three studies also documented maximum levels, while seven articles reported the submaximal intensity range. The shortest intervention period implemented in the reports was 6 weeks, with 12 weeks being the longest.

### Quality and bias analyses

**Fig. 2:**
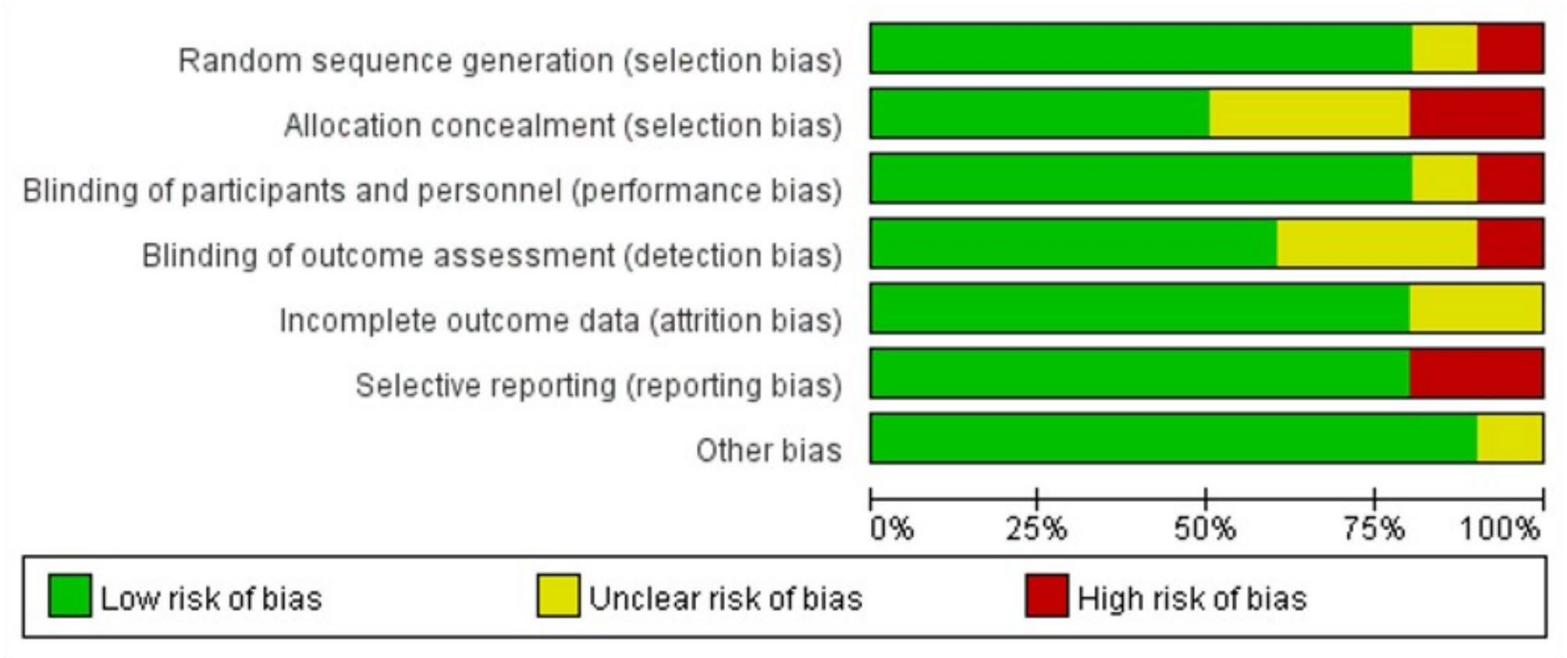
Analysis of risk of bias according to the Cochrane collaboration guideline

Figure 2 illustrates the methodological quality and potential risk of bias results of the articles reviewed. The overall quality of the articles was relatively significant. Meanwhile, the publications had high, unclear, and low bias risks at 10.0%, 15.7%, and 74.3%, respectively (Fig. 2).

### Quantitative synthesis

**Fig. 3:**
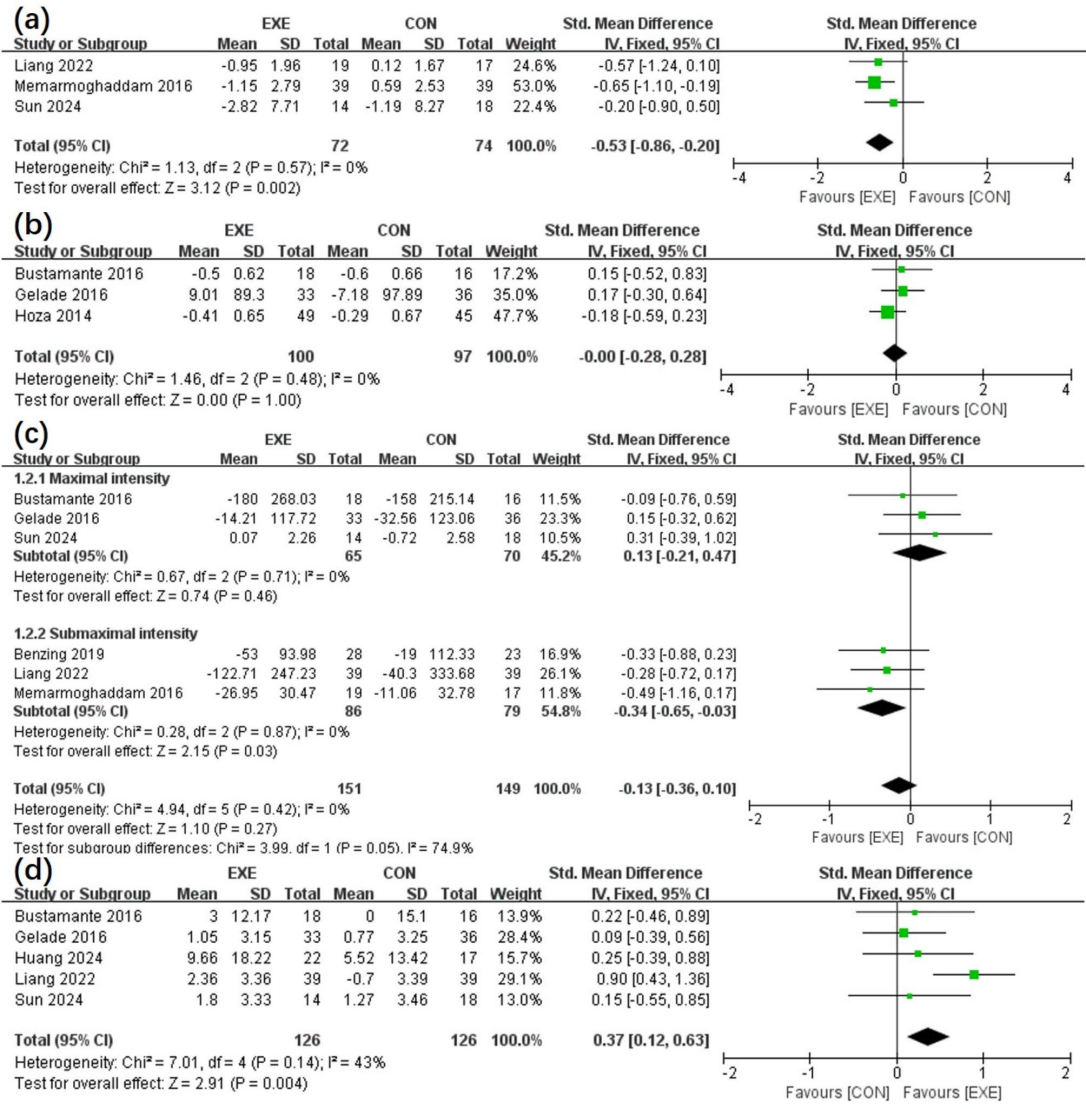
Forest plot portraying the effects of EXE vs. CON intervention on the cognition (**a**), inattention (**b**), inhibitory control (**c**) and working memory (**d**)

Comparisons of the effects between the EXE and CON groups on cognition [17, 18, 20] and inattention [6, 14, 21] of three articles reviewed are demonstrated in Fig. 3(a) and (b). The results indicated that the participants in the EXE category recorded improved cognition [SMD, − 0.53 (− 0.86, − 0.20), *p* < 0.05, I^2^ = 0%, p for heterogeneity = 0.57] than the CON group. Nevertheless, no statistically significant differences were observed concerning inattention [SMD, − 0.00 (− 0.28, 0.28), *p* = 1.00, I^2^ = 0%, p for heterogeneity = 0.48].

The effects of EXE and CON interventions on inhibitory control were compared for six of the selected articles [6, 17, 18, 20–22] [Fig. 3(c)]. No statistically notable variations were documented [SMD, − 0.13 (− 0.36, 0.10), *p* = 0.27, I^2^ = 0%, p for heterogeneity = 0.42]. Figure 3(d) illustrates the working memory comparisons of five reviewed publications [6, 17, 18, 21, 23]. Participants in the EXE group enhanced working memory [SMD, 0.37 (0.12, 0.63) *p* < 0.05, I^2^ = 0%, p for heterogeneity = 0.14] than the CON group.

Subgroup evaluations indicated that interventions with maximal exercise intensity did not considerably affect the inhibitory control levels of the participants [SMD, 0.13 (− 0.21, 0.47), *p* = 0.46, I^2^ = 0%, p for heterogeneity = 0.71]. Conversely, submaximal intensity exercise improved inhibition regulation levels significantly [SMD, − 0.34 (− 0.65, − 0.03), *p* < 0.05, I^2^ = 0%, p for heterogeneity = 0.87].

**Fig. 4:**
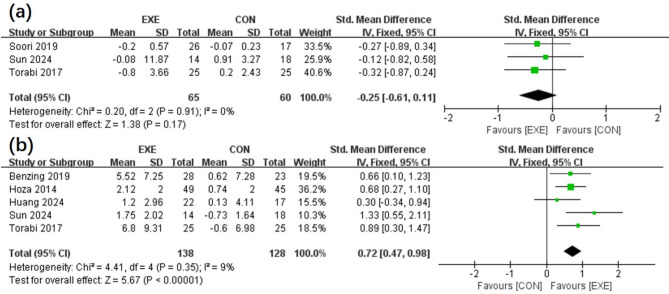
Forest plot portraying the effects of EXE vs. CON intervention on BMI (**a**) and motor ability (**b**)

The body mass index (BMI) of EXE and CON groups in three articles [17, 24, 25] were compared [Fig. 4(a)]. Meanwhile, the motor ability data from five publications [14, 17, 22, 23, 25] are illustrated in Fig. 4(b). The meta-analysis revealed that increased motor ability [SMD, 0.72 (0.47, 0.98), *p* < 0.05, I^2^ = 9%, p for heterogeneity = 0.35) benefited the EXE group more than the CON category. Nonetheless, statistically substantial differences in BMI were not documented (SMD, -0.25 [-0.61, 0.11], *p* = 0.17, I^2^ = 0%, p for heterogeneity = 0.91).

### Bias assessment

**Fig. 5:**
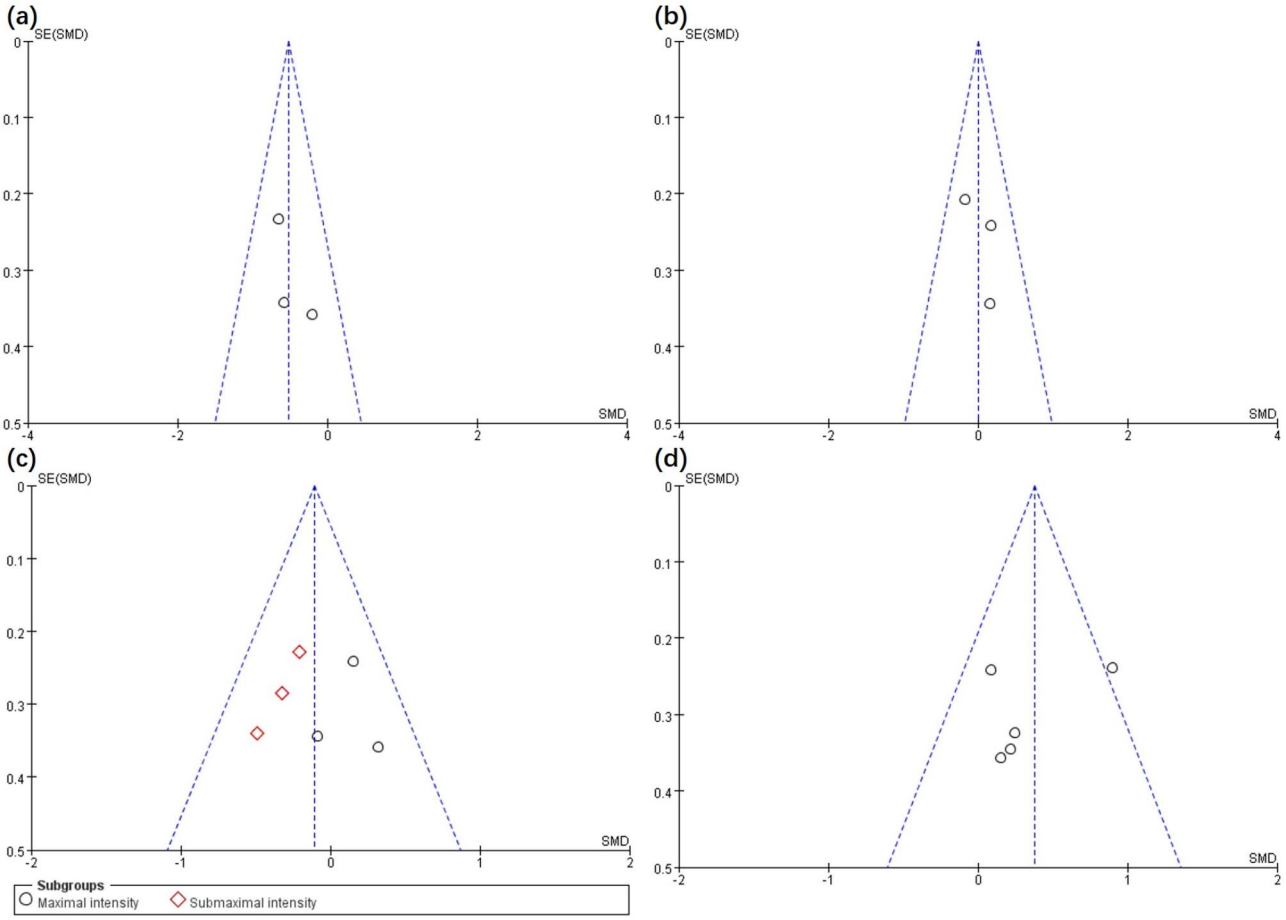
Funnel plot of publication bias in the EX vs. CU intervention for the cognition (**a**), inattention (**b**), inhibitory control (**c**) and working memory (**d**)

**Fig. 6:**
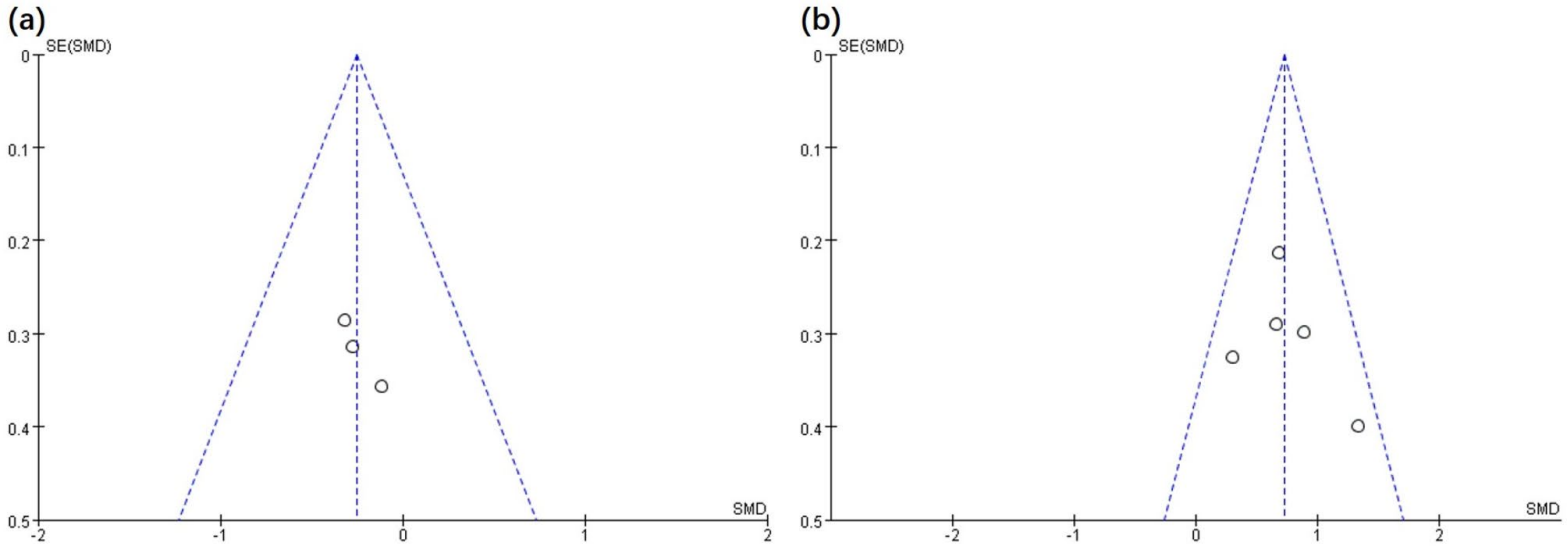
Funnel plot of publication bias in the EX vs. CU intervention for the BMI (**a**) and motor ability (**b**)

Although only 10 articles were reviewed in this study, the number approached the minimum requirement for funnel plot implementation. Although publication bias might only be observed to some extent, minor sample publication bias has been reported [27]. Based on Figs. 5 and 6 and a left-right symmetrical distribution indicating a low probability of publication bias was documented for the articles reviewed.

### Sensitivity analysis

According to the results, no significant alterations in each group were recorded following analysis type and impact size modifications and individual studies exclusion. The sensitivity analysis revealed that the findings were reliable.

## Discussion

Central executive function components form the executive function infrastructure, including working memory and inhibitory control [28]. Executive function regulates basal cognition through top-down higher mental processes essential for attaining goal-directed adaptive behaviours and maintaining attention [29]. Major deficits in executive function are the predominant symptoms of ADHD, with most patients exhibiting one or more deficiencies [30]. Inhibitory control, cognitive flexibility, and working memory are the three core functions central to ADHD patients’ psychological and behavioural characteristics, contributing to their performance in school, work, and social settings [31].

The present review evaluated the effects of vigorous-intensity activities on working memory and inhibitory control in children with ADHD. According to the results, vigorous physical activities primarily improved working memory in children suffering from the disorder significantly. Nonetheless, the activities had no considerable influence on inhibitory control. Furthermore, subgroup evaluations revealed that submaximal rather than maximal intensity exercises effectively increased inhibitory control in children with ADHD. The cognitive and motor abilities of children ADHD patients were also enhanced with vigorous-intensity exercise interventions. Nevertheless, the treatment did not affect attention and BMI. The findings indicated that vigorous-intensity activities are a potential non-pharmacological approach to improve ADHD symptoms in children.

An investigation on the effects of physical activities on executive function in ADHD patients between 7 and 24 years old found that exercise, particularly vigorous intensity exercise, significantly improved the executive function of ADHD patients, supporting the findings of this review. Nevertheless, the meta-analysis investigation did not distinguish between maximal and submaximal intensity exercises. The effects of the interventions on BMI and exercise capacity were also not evaluated.

The study results revealed that submaximal intensity exercise protocol had substantial benefits on inhibitory management in children ADHD patients. Although the maximal intensity physical activities exhibited an improving inhibitory control function trend, the evidence provided insignificant differences. Individuals suffering from ADHD are fundamentally impulsive, hyperactive, or intolerant [32]. Consequently, patients with the illness typically find regular exercise therapy challenging [33]. Moreover, age and ADHD attributes make it almost impossible for the patients to adhere to long daily physical activities [33]. High-intensity and low-duration anaerobic exercise therapy could provide a solution [34].

Short-term vigorous-intensity interval exercise substantially increased the inhibitory levels of ADHD adolescents [35]. Nonetheless, the mechanisms are unclear in child patients. A report hypothesised that vigorous-intensity physical activities could contribute to the neural transmission of catecholamines [36]. In the long term, the intervention might effectively promote prefrontal cortical development and increase the hippocampus or the total grey and white matter volume of the brain. Moreover, the information transmission and cerebral blood flow between different brain regions, oxygen supply to the brain frontal lobes, and nerve cell regenerative capacity are enhanced, ultimately improving inhibitory regulation in the patients [16].

Walking memory is a crucial component of executive function. Children with ADHD frequently exhibit walking memory core mechanism deficits, which is memory updating [37]. The patients are also typically unable to effectively manage and allocate cognitive resources [37]. The data in this review indicated improved working memory in children with ADHD prescribed with vigorous intensity exercises. The finding also supported previous reports. For instance, Huang et al. [38] administered an eight-week rope-skipping intervention to children with ADHD. The intensity of the activity ranged between 64% and 95% HR_max_. After eight weeks, the participants exhibited significantly enhanced working memory.

The mechanism of vigorous-intensity exercises in improving walking memory might be related to neurotransmitters such as catecholamines. Several neuroimaging reports have also demonstrated that vigorous-intensity aerobic activities could alter brain plasticity, improving walking memory in children suffering from ADHD. Moreover, functional magnetic resonance imaging investigations indicated after an eight-week exercise intervention, the activation levels of brain regions associated with walking in children with ADHD were substantially improved, including the left middle and right superior frontal gyrus and the right posterior cingulate cortex [39].

Functional near-infrared spectroscopy investigations noted that aerobic exercises contribute to known brain hypoactive area regulation of individuals with ADHD, including temporal and parietal lobe junctions and middle and lower frontal lobe sections [40] Resting electroencephalogram findings also reported that following eight weeks of aerobic exercise, children with ADHD exhibited a reduced θ/α ratio in brain areas concerning walking memory, such as the frontal and central brain regions [38]. Overall, the mechanisms by which vigorous intensity exercise enhances executive functions, including inhibitory control and working memory, are attributable to improved brain internal environment.

The present study has numerous limitations. Only 10 articles were selected for review. The severity of ADHD symptoms in the participants was also inconsistent, which might inaccurately represent the entire population. Moreover, different research objectives led to incomparable forms of exercise interventions, limiting horizontal comparisons. Accordingly, future works should consider incorporating more reports to enhance the credibility of the results. Dose-dependent relationship between exercise and ADHD symptom improvement should also be considered to determine the optimal exercise intensity to enhance the physiological and psychological functions of children with ADHD. In the exercise rehabilitation practice for children with ADHD, submaximal intensity exercise seems to be a safer and more effective choice.

## Conclusion

Based on the systematic meta-analysis results, vigorous-intensity exercises have effective working memory, cognitive function, and motor ability-increasing effects on children with ADHD. Furthermore, Submaximal intensity exercise can effectively improve control inhibition in children with ADHD. The interventions could present a promising non-pharmacological strategy to improve ADHD symptoms in children through diverse exercise prescription options.

## Electronic supplementary material

Below is the link to the electronic supplementary material.

Supplementary Material 1

## Data Availability

If necessary, it can be obtained from the corresponding author.

## Abbreviations

ADHD: Attention deficit hyperactivity disorder
CI: Confidence intervals
CON: Control group
EXE: Exercise group
RCT: Randomised controlled trial
SMD: Standardised mean difference
