# Supplementary material for: Effect of vigorous-intensity exercise on the working memory and inhibitory control among children with attention deficit hyperactivity disorder: a systematic review and meta-analysis
Source: Ital J Pediatr. 2025 Mar 28;51:104. doi: 10.1186/s13052-025-01924-w (PMC11951569; doi:10.1186/s13052-025-01924-w)
Supplement: Supplementary file 1 — Supplementary Material 1 [file 13052_2025_1924_MOESM1_ESM.docx]

**Appendix A**

Search strategy

| Databases | Search strategy | Result  (Approximately) |
| --- | --- | --- |
| Scopus | #1: Title-Abs-Key (Children or Adolescents)  #2: Title-Abs-Key (Exercise or Training)  #3: Title-Abs-Key (ADHD or Attention deficit hyperactivity disorder)  #4: Title-Abs-Key (High intensity or Vigorous intensity)  #5: #1 and #2 and #3 and #4  Limiters - Published Date: 20041101-20241101 | 2,243,765  1,993,458  53,267  345,781  29 |
| Pubmed | #1: [Title/Abstract] Children or Adolescents  #2: [Title/Abstract] Exercise or Training  #3: [Title/Abstract] ADHD or Attention deficit hyperactivity disorder  #4: [Title/Abstract] High intensity or Vigorous intensity  #5: #1 and #2 and #3 and #4  Filters: Publication date from 2004/11/01 to 2024/11/01 | 4,515,529  3,235,767  52,223  204,414  19 |
| Web of Science | #1: TOPIC: (Children or Adolescents)  #2: TOPIC: (Exercise or Training)  #3: TOPIC: (ADHD or Attention deficit hyperactivity disorder)  #4: TOPIC: (High intensity or Vigorous intensity)  #5: #1 and #2 and #3 and #4  Refined by: PUBLICATION YEARS: (20241101-20041101)  Indexes=SCI-EXPANDED, SSCI, CCR-EXPANDED, | 1,468,913  1,199,163  47,893  406,032  53 |
| EBSCO | #1: Abstract: (Children or Adolescents)  #2: Abstract: (Exercise or Training)  #3: Abstract: (ADHD or Attention deficit hyperactivity disorder)  #4: Abstract: (High intensity or Vigorous intensity)  #5: #1 and #2 and #3 and #4  Year: 20041101-20241101 | 2,762,167  1,876,257  4,197  311,285  36 |
